# Procalcitonin, pyuria and proadrenomedullin in the management of urinary tract infections – ‘triple p in uti’: study protocol for a randomized controlled trial

**DOI:** 10.1186/1745-6215-14-84

**Published:** 2013-03-22

**Authors:** Daniel Drozdov, Anja Thomer, Marc Meili, Stefanie Schwarz, Rita Bossart Kouegbe, Katharina Regez, Merih Guglielmetti, Ursula Schild, Antoinette Conca, Petra Schäfer, Barbara Reutlinger, Cornelia Ottiger, Florian Buchkremer, Alexander Litke, Philipp Schuetz, Andreas Huber, Ulrich Bürgi, Christoph A Fux, Andreas Bock, Beat Müller, Werner C Albrich

**Affiliations:** 1 Medical University Department, University of Basel, Kantonsspital Aarau, Tellstrasse, Aarau, 5001, Switzerland; 2 Division of Infectious Diseases, Kantonsspital Aarau, Tellstrasse, Aarau, 5001, Switzerland; 3 Division of Nephrology, Kantonsspital Aarau, Tellstrasse, Aarau, 5001, Switzerland; 4 Department of Clinical Nursing Science, Kantonsspital Aarau, Tellstrasse, Aarau, 5001, Switzerland; 5 Department of Emergency Medicine, Kantonsspital Aarau, Tellstrasse, Aarau, 5001, Switzerland; 6 Department of Laboratory Medicine, Kantonsspital Aarau, Tellstrasse, Aarau, 5001, Switzerland; 7 Department of Infectious Diseases, Kantonsspital St Gallen, Rorschacher Strasse 95, St Gallen, 9007, Switzerland

## Abstract

**Background:**

Urinary tract infections (UTIs) are among the most common infectious diseases and drivers of antibiotic use and in-hospital days. A reduction of antibiotic use potentially lowers the risk of antibiotic resistance. An early and adequate risk assessment combining medical, biopsychosocial and functional risk scores has the potential to optimize site-of-care decisions and thus allocation of limited health-care resources. The aim of this factorial design study is twofold: first, for Intervention A, it investigates antibiotic exposure of patients treated with a protocol based on the type of UTI, procalcitonin (PCT) and pyuria. Second, for Intervention B, it investigates the usefulness of the prognostic biomarker proadrenomedullin (ProADM) integrated into an interdisciplinary assessment bundle for site-of-care decisions.

**Methods and design:**

This randomized controlled open-label trial has a factorial design (2 × 2). Randomization of patients will be based on a pre-specified computer-generated randomization list and independent for the two interventions. Adults with UTI presenting to the emergency department (ED) will be screened and enrolled after providing informed consent.

For our first Intervention (A), we developed a protocol based on previous observational research to recommend initiation and duration of antibiotic use based on the clinical presentation of UTI, pyuria and PCT levels. For our second intervention (B), an algorithm was developed to support site-of care decisions based on the prognostic marker ProADM and distinct nursing factors on days 1 and 3. Both interventions will be compared with a control group conforming to the guidelines.

The primary endpoints for the two interventions will be: (A) overall exposure to antibiotics and (B) length of physician-led hospitalization within a follow-up of 30 days. Endpoints are assessed at discharge from hospital, and 30 and 90 days after admission. We plan to screen 300 patients and enroll 250 for an anticipated estimated loss of follow-up of 20%. This will provide adequate power for the two interventions.

**Discussion:**

This trial investigates two strategies for improved individualized medical care in patients with UTI. The minimally effective duration of antibiotic therapy is not known for UTIs, which is important for reducing the selection pressure for antibiotic resistance, costs and drug-related side effects. Triage decisions must be improved to reflect the true medical, biopsychosocial and functional risks in order to allocate patients to the most appropriate care setting and reduce hospital-acquired disability.

**Trial registration:**

Trial registration number: ISRCTN13663741

## Background

Urinary tract infections (UTIs) include acute cystitis (involving the lower urinary tract) and acute pyelonephritis (involving the upper urinary tract). If a structural or functional abnormality is present, the UTI is considered ‘complicated’, implying a higher risk of encountering antimicrobial resistance and therapy failure [1]. *Escherichia coli* has been identified as the most common causative pathogen in uncomplicated (75%) as well as in complicated UTIs (40% to 50%) [2].

Current empiric antibiotic therapy schemes for UTIs are based on guidelines that largely reflect expert opinion [3,4]. There are few intervention studies comparing different durations of antibiotic therapy [5-7]. Considering the emerging antibiotic resistance of uropathogens [2,8,9] and the growing awareness of the epidemiological side effects of antibiotics [10], efforts aimed at improvements should be intensified.

There is increasing interest in biomarkers as diagnostic and prognostic factors in infectious diseases since they reflect the host’s response and are objective, dynamic and easily measurable. In particular, the biomarker procalcitonin (PCT) has been thoroughly investigated and has been proven to be a useful tool in managing the antibiotic therapy of bacterial infections of the respiratory tract [11,12]. In a previous multicenter study of patients with sepsis admitted to French intensive care units (ICUs), a PCT-guided strategy for treating bacterial infections safely reduced antibiotic exposure compared to present guidelines [13]. Of note, 7% of the enrolled patients had a UTI as their source of infection. PCT primarily indicates systemic infections. Thus, in patients with UTIs, PCT likely needs to be combined with inflammatory surrogates of local infection [14] such as pyuria or a urinary protein profile. The normalization of pyuria or a drop of at least 90% in urinary leukocytes correlated with a successful outcome of UTI therapy [15]. The tubular injury marker, the α1-microglobulin/creatinine ratio, has a high specificity and sensitivity in distinguishing pyelonephritis from cystitis and is positively correlated with baseline C-reactive protein (CRP) levels [16-18].

Radiologic evaluation of the urinary tract in patients with a febrile UTI is frequently performed. The European Association of Urology recommends ultrasound evaluation to rule out obstruction and renal stone disease. Additional radiologic testing (for example, computed tomography, excretory urogram or 99mTechnetium-dimercaptosuccinic acid scan) ‘should be considered’ if fever persists after 72 h of treatment [19]. A more selective approach to imaging using a clinical prediction rule has been advocated [20]. Contrast-enhanced ultrasound (CEUS) is a promising method for detecting acute pyelonephritis [21]. Its sensitivity and specificity might approach that of computed tomography [22], but the available data are very limited.

In adults ≥65 years, UTIs are the second most common cause for hospitalization among infectious diseases [23] with an annual cost of approximately $1.6 billion in the USA alone [24]. Older patients have a high risk of becoming functionally impaired or lose their self-care abilities if they are hospitalized [25], possibly leading to further inpatient treatment or an increased length of stay (LOS) whereas outpatient treatment is less expensive [26] and carries a lower risk of subsequent disability. Particularly for lower respiratory tract infections (LRTIs), many patients are hospitalized for fear of medical complications [27] or for medical co-morbidities, functional and psychosocial reasons, despite a low-risk classification according to clinical severity scores [26,28]. Accordingly, one-fifth of low-risk inpatients with community acquired pneumonia (CAP) do not have any contraindications for outpatient treatment or identifiable risk factors for hospitalization [28]. Multidisciplinary assessment in order to triage to the most appropriate care setting [29-31], as well as innovative pathway bundles are important in reducing the hospitalization rate, LOS, antibiotic use and overall costs while achieving similar quality of life and patient outcomes as explored for LRTI [32,33]. For UTIs equivalent studies are lacking so far, but as evident in daily clinical practice, improved triage for these infections is urgently needed. For LRTI we showed that ProADM was the most accurate biomarker for prognostic assessment [31,34,35]. As it is not specific for LRTIs, we propose that ProADM will be an excellent marker for prediction of re-hospitalization and death in UTIs [36].

## Methods

**Figure 1:**
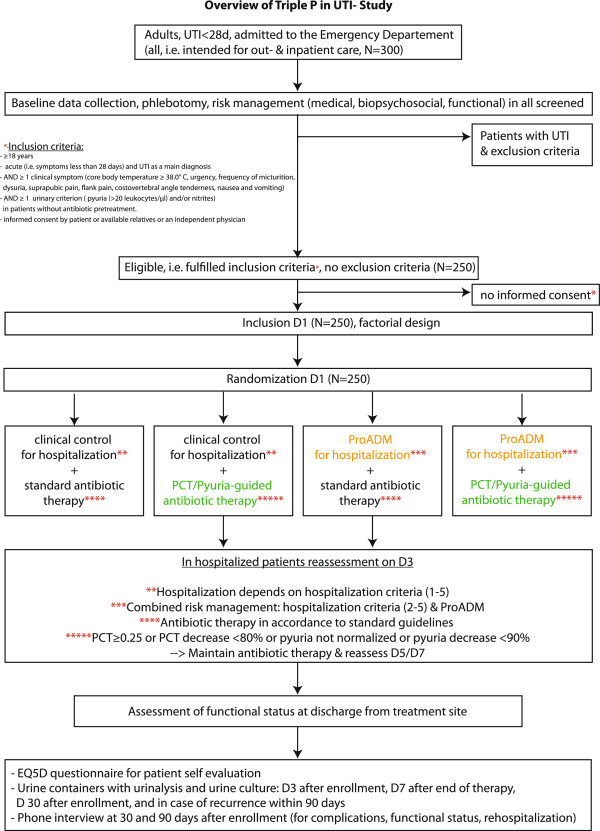
**Trial overview.** D1: day 1; D3: day 3; D5: day 5; D7: day 7; D30: day 30.

This is a randomized controlled open-label trial using a two-by-two factorial study design with two independent internet-based 1:1 randomizations (one each for the antibiotic part and one for the triage part, respectively) (Figure 1). The objectives are first, for Intervention A, to analyze the efficacy and safety of a PCT- and pyuria-guided antibiotic therapy in individualizing and reducing the duration of antibiotic treatment compared to the guidelines [4,10,19,37] and second, for Intervention B, to test the impact on LOS of ProADM-enhanced triage decisions compared to standard care. Both interventions are for a high-intensity implementation of interdisciplinary risk assessment for triage in patients with UTIs presenting to an ED. LOS is defined as the number of days of physician-led hospitalization until discharge to ambulatory or nurse-led care.

The trial is supervised by an independent safety monitoring board, which is not involved in the design and conduct of the trial. The board consists of three experts in internal medicine, infectious diseases, nephrology and epidemiology, respectively.

Ethical approval for this trial, which is in compliance with the Helsinki Declaration and in agreement with the CONSORT statement [38], has been obtained from the local ethical committee. The institutional review board of the Canton Aargau approved the study protocol. All participating patients or their relatives must give written informed consent.

### Setting

In April 2012, we started to screen all adult patients with a suspected UTI admitted to the ED of the public Cantonal Hospital of Aarau, a tertiary-care 600-bed hospital in northern Switzerland. We expect to finish the study by December 2014.

Physicians in charge of patient recruitment and inclusion and nursing staff attended a structured seminar on evidence-based guidelines for the management of patients with UTIs, the details of the protocol, the rationale and the design of the trial and the online website.

### Participants

Inclusion criteria for patients are age ≥18 years and admission from the community or a nursing home with a main diagnosis of acute UTI (that is, symptoms less than 28 days). Exclusion criteria are other infections that require an antibiotic therapy, pre-treatment with antibiotics within the last 48 h, pregnancy, prostatitis (defined as pain on digital rectal examination or a Prostate-speicifc antigen (PSA) > 4 ng/ml or PSA > 2× baseline) presence of implanted foreign bodies in the urinary tract or urinary catheters, endovascular prostheses or foreign bodies, non-endovascular prostheses or foreign bodies within 6 months after implantation, insufficient language skills with no possibility for translation, foreseeable non-compliance for follow-up (for example, current drug abuse), severe immunodeficiency (neutrophiles <500/μL, CD4 cells <350/μL in patients with HIV infection, leukemia, lymphoma, myeloma, cytotoxic medications, hemodialysis, transplant patients) or severe medical co-morbidity with imminent death.

### Definitions

UTI is defined by at least one clinical symptom (core body temperature ≥38.0°C, urinary urgency, polyuria, dysuria, suprapubic pain, flank pain, costovertebral angle tenderness, nausea and vomiting) and one urinary criterion (pyuria >20 leukocytes/μl [39] and/or nitrites). A UTI is defined as febrile UTI/pyelonephritis if there is flank pain, costovertebral angle tenderness or a body temperature ≥38°C, and otherwise as simple UTI. A UTI is defined as complicated if any of the following criteria are fulfilled: male gender, age >70 years, symptoms >7 days, recent antibiotic therapy (within 30 days), recurrent UTIs (two or more during the last 6 months or three during the last 12 months), recent urologic intervention (30 days), functional or anatomic abnormality, diabetes mellitus or immunosuppressive therapy; otherwise UTI is considered uncomplicated for example, young non-pregnant women.

### Randomization

Randomization of patients to either intervention is based on a pre-specified computer-generated randomization list and concealed by using a centralized password-secured website. Based on the factorial design, the two randomizations are independent.

### Interventions

With enrollment of each patient, the physician in charge is reminded of the processes of care in the ED. The recommendations include the choice of antibiotic therapy and duration as well as site-of-care decisions, together with reminders about biopsychosocial and functional overruling criteria and medical risk.

#### Intervention A

**Figure 2:**
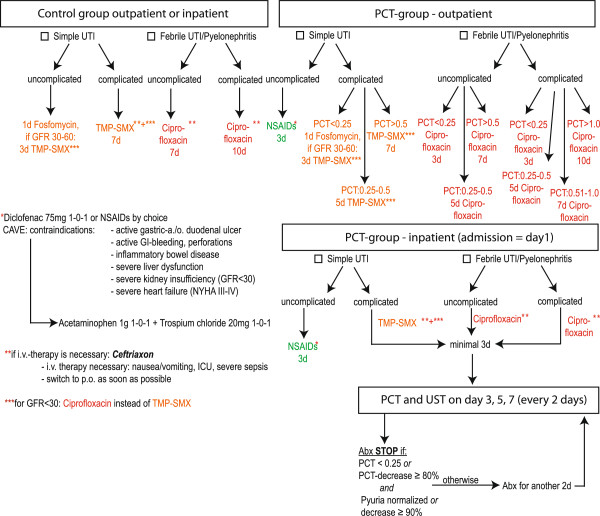
**Individualized antibiotic guidance by PCT and pyuria.** The algorithm for diagnostic purposes is shown. If there is any concern about microbiologic resistance, therapy extension is considered. For proven microbiologic resistance therapy is adjusted. Remaining antibiotic treatment after patient discharge is guided by last PCT value to result in total antibiotic duration in analogy to outpatient treatment. Abx: antibiotics; GFR: estimated glomerular filtration rate with MDRD formula; ICU: intensive care unit; NSAIDs: nonsteroidal anti-inflammatory drugs; PCT: procalcitonin; TMP-SMX: trimethoprim-sulfamethoxazole; UST: urinalysis; UTI: urinary tract infection.

Appropriate antibiotics and minimal duration of antibiotic treatment are based on recent guidelines [10] after local adaptation by a panel of experts. PCT is used to guide antibiotic treatment (Figure 2). We use fosfomycin (3 g single dose) [40] and trimethoprim-sulfamethoxazole (800/160 mg twice daily) for simple UTIs. Furthermore we use ciprofloxacin orally (500 mg twice daily or 250 mg twice daily if the estimated glomerular filtration rate <30 ml/min/1.73 m2) or ceftriaxone intravenously (2 g daily) for febrile UTIs/pyelonephritis. If known, previous antibiotic resistant profiles are considered. If there is a non-susceptible urine pathogen in the current urine culture the antibiotic therapy is adjusted. In inpatients, antibiotic duration is based on absolute values of PCT and relative decreases of PCT levels and pyuria. In outpatients, the antibiotic duration is based on absolute values of PCT only. PCT and pyuria are measured on admission, and, in hospitalized patients, every other day as long as the patient receives antibiotics for the UTI. Pyuria criteria are derived from local data on female patients with UTIs [15]. PCT cutoffs are derived from data from our recent observational study and after extrapolation from patients with LRTIs and sepsis [36,41,42]. Patients with uncomplicated simple UTIs in the PCT group receive NSAIDs regardless of PCT values for symptom resolution given emerging data [43,44]. In the control group, antibiotic durations are recommended based on guidelines (Figure 2).

Since failure of pyuria to decrease at least 90% within 24 h of antibiotic therapy was associated with treatment failure in patients with lower UTIs [15], we collect a urine specimen after 48 h (day 3) of antibiotic therapy in all patients. If patients are already at home at this time, they are given a container for urine to be returned for a urinalysis including determination of pyuria (Figure 1). We also collect urine for urinalysis and urine culture on day 7 after end of therapy, day 30 after enrollment, and in case of recurrence within 90 days. The patients are instructed to collect clean midstream urine into sterile urine containers. A vacuum is used to transfer the urine from the container to special urine tubes: BD Vacutainer® Plus Preservative Tube with ethyl paraben, sodium propionate and chlorhexidine preservatives for urinalysis and BD Vacutainer® Plus C&S Preservative Tube with boric acid, sodium formate and sodium borate for urine culture. These urine specimens are sent by priority mail in prestamped envelopes immediately after collection. Priority mail requires typically less than 24 h (maximum 48 h) to arrive at our hospital laboratory.

#### Intervention B

**Figure 3:**
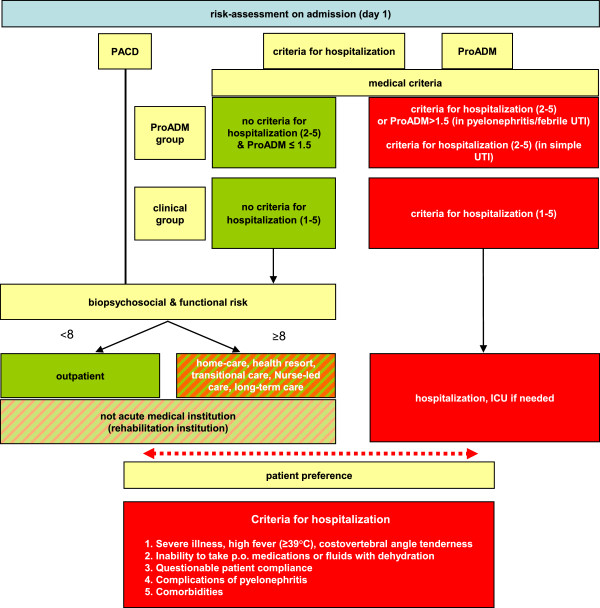
**Risk assessment on admission - site-of-care decisions (ProADM on admission).** ICU: intensive care unit; PACD: post-acute care discharge score; ProADM: proadrenomedullin; UTI: urinary tract infection.

The triage decision in the ED is based on an interdisciplinary biopsychosocial and functional risk assessment by the treating physician (for medical risk) and the ED nursing team (for biopsychosocial risk) (Figure 3). Hospitalization criteria in the control group include: 1. severe illness, high fever (≥39°C), costovertebral angle tenderness or flank pain; 2. inability to take oral medications or fluids; dehydration; 3. questionable patient compliance; 4. complications of pyelonephritis and 5. co-morbidities. In the ProADM group, the subjective admission criterion 1 (severe illness, high fever, costovertebral angle tenderness, severely impaired health) is omitted (simple UTI) or replaced by objective ProADM cutoffs for patients with febrile UTI/pyelonephritis. In patients with simple UTI, we do not expect the ProADM levels to provide sufficient prognostic information and therefore do not include ProADM in their admission criteria. A standardized score (the post-acute care discharge score, PACD) [45] is used to stratify all patients as low or high risk for subsequent rehabilitation and care. The final triage decision is a consensus by physicians, nurses and patient (or relatives). If a consensus cannot be reached, the patient’s preference has priority.

**Figure 4:**
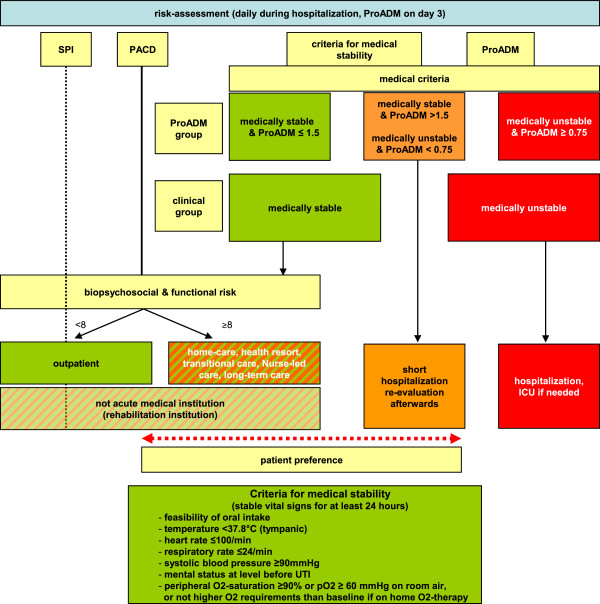
**Risk assessment during hospitalization - site-of-care decisions (ProADM on day 3).** ICU: intensive care unit; PACD: post-acute care discharge score; SPI: *Selbstpflegeindex* self-care deficit score; UTI: urinary tract infection.

The clinical risk assessment of both doctors and nurses is standardized using medical stability criteria [46] and biopsychosocial and functional scores (*Selbstpflegeindex* (SPI) self-care index [45,47]) and is used to assess the suitability of patients for discharge from the hospital (Figure 4). The care and risk assessment bundle is implemented in both groups using behavioral and communication strategies proven to be highly effective [48].

Levels of ProADM are measured on admission and on day 3. ProADM cutoff tertiles derived from patients with LRTI will be used for severity assessment [41]: <0.75 nmol/l; 0.75 to 1.5 nmol/l; >1.5 nmol/l. Physicians receive detailed information on how to interpret ProADM cutoffs and about the results from previous trials when ProADM was used in patients with LRTI [12,29,31,34,35,49-51]. ProADM is measured in an ethylenediaminetetraacetic acid (EDTA) plasma by a sandwich immunoassay (MR-ProADM, BRAHMS AG, Hennigsdorf, Germany) with an analytical detection limit of 0.08 nmol/l and a functional assay sensitivity of 0.12 nmol/l. It is available within 1.5 h upon ordering (24 h a day, daily).

Reassessment of the possibility for discharge from the acute-care hospital is based upon reaching medical stability at any time plus appropriate ProADM levels with particular emphasis on assessment on day 3 (Figure 4). On day 3, the treating physicians receive recommendations regarding discharge according to the clinical stability algorithm. Guidelines are displayed on handouts given to the treating physicians. Algorithm compliance is controlled by the study team and reasons for non-compliance are documented.

Discharge planning based on the biopsychosocial risk at admission (PACD ≥ 8) is initiated immediately and any need for action discussed at each round.

Patients with low medical risk or medically stabilized but high biopsychosocial requirements can be triaged to nurse-led care. The nursing staff is in charge of further management with medical input only at further request or if there is a medical deterioration.

Medically stable patients not fulfilling medical criteria and biopsychosocial, functional and organizational overruling criteria (see section “Overruling criteria for antibiotic treatment and hospitalization”) are considered suitable for discharge to home. Patients meeting biopsychosocial or functional overruling criteria and organizational criteria (waiting for a transfer to the non-acute setting) are referred to non-acute medical care (nurse-led care, health resort, rehabilitation, transitional care or long-term care) (Figure 4).

### Overruling criteria for antibiotic treatment and hospitalization

Medical overruling criteria for prolonged antibiotic therapy

1. Admission to ICU

2. Life-threatening co-morbidity

3. Complications: abscess, papillary necrosis, emphysematous pyelonephritis, acute renal failure (GFR < 30 ml/min or creatinine increase ≥50% from baseline level)

Medical overruling criteria for hospitalization

1. Admission to ICU

2. Life-threatening co-morbidity

3. Complications: pyelonephritis, acute renal failure (GFR < 30 ml/min or creatinine increase ≥50% from baseline level)

4. Antibiotics or hospitalization (regardless of indication) within the last week

5. Confusion, delirium or intravenous drug abuse

Biopsychosocial and functional overruling criteria for hospitalization

1. Dementia, recurrent falls, pressure ulcer and inability to reliably take medications

2. SPI score <32 (during hospitalization)

3. Deficit of mobility

Organizational overruling criteria for hospitalization

1. Waiting for results that may alter the management, lab controls, investigations or therapy

2. Waiting for relocation to a non-acute medical care facility

3. Weekend or night

Preferences of the patient and the relatives

1. Patient and relatives concern about safety at home

2. Lack of nursing care

3. Financial reasons

#### Measurements

Urine sediment analysis, urine culture with an antibiotic resistance profile and urinary protein profile (α1-microglobulin/creatinine ratio) are performed on admission. In patients with a diagnosis of febrile UTI/pyelonephritis, blood cultures are taken prior to administration of antibiotics. These patients also receive an ultrasound evaluation of the urinary tract, including Doppler examination as well as a CEUS examination of both kidneys.

Chargeable and effective costs will be gathered from the finance and controlling departments to calculate costs per patient from study entry until discharge to a non-hospital setting. Data on outcome and complications will be collected by the study team.

All patients are called by members of the study team (who are unaware of the randomization group) on days 30 and 90 after randomization to inquire about clinical recurrences, antibiotic side effects and the preparedness for discharge [52] using a questionnaire with a ten-point Likert scale response format. Interviews are standardized according to a protocol and interviewers receive formal introductory training.

Physicians in this trial know that their behavior is monitored. Since it is not feasible to have ProADM and PCT values blinded in the control groups, however, algorithm compliance is reinforced according to assigned groups by the study team.

Both contrast-enhanced ultrasound criteria and urinary protein profiles are used together with urine and clinical criteria and biomarker levels for the final classification as upper or lower UTI. For final analysis, culture confirmation will be required (Table 1).

**Table 1:** Urine culture cutoffs for significance

**Diagnosis**	**Criteria**
Uncomplicated, simple UTI	≥10^3^ cfu/ml
Uncomplicated febrile UTI/pyelonephritis	≥10^4^ cfu/ml
Complicated UTI (simple or febrile UTI/pyelonephritis)	
in women	≥10^5^ cfu/ml
in men	≥10^4^ cfu/ml
in straight catheter urine	≥10^4^ cfu/ml

cfu: colony-forming unit; UTI: urinary tract infection.

Healthcare-related quality of life on the day of discharge is measured with the EQ-5D questionnaire. EQ-5D includes the 15-item EQ-5D self-classifier, which assesses the health-related quality of life among five dimensions and the EQVas, which obtains a self-rating of the current health-related quality of life [53].

### Outcomes and adverse events

The primary endpoint for Intervention A is overall exposure to antibiotics in the PCT and pyuria guidance group compared to the control group. Endpoints are assessed at discharge from hospital, and 30 and 90 days after admission. As secondary endpoints, we will compare the PCT-pyuria group with the control group regarding:

A. Clinical and microbiological cure 7 days after end of therapy.

B. 30-day rate of clinical and microbiological recurrence.

C. 90-day rate of clinical and microbiological recurrence.

D. Antibiotic-associated side effects.

Regardless of group assignment, we will investigate:

E. The relationships of pyuria, urine culture cutoffs and urinary α1-microglobulin/creatinine ratio with the presence of pyelonephritis and level of biomarkers (CRP, PCT, ProADM, and other biomarkers such as urea, natriuretic peptides (ANP, BNP), copeptin, endothelin, apoprotein A1).

F. Correlation between sonographic signs of pyelonephritis and diagnosis based on urine culture cutoffs, clinical criteria and biomarkers.

Our hypothesis for the secondary endpoints A to C is that there is no difference between the two study groups. Our hypothesis for endpoints E and F is that urinary markers and contrast-enhanced ultrasound correlate with systemic biomarkers and diagnosis of pyelonephritis.

For the primary endpoint for Intervention B, we assess the length of physician-led hospitalization in the ProADM-guided triage group compared to the control group within a follow-up of 90 days. As secondary endpoints, we will compare the ProADM group with the control group regarding:

A. resource utilization, defined as the length of index hospitalization (physician- and/or nurse-led); rate and length of re-hospitalization; rate of general practitioner visits; rate of medical investigations and interventions related to UTI and overall, rate of treatment changes related to UTI, for example, antibiotic escalations. The hypothesis is that there are no excess re-hospitalizations or visits to the GP while there are fewer treatment changes and medical investigations in the ProADM group.

B. Proportion of patients triaged according to the risk assessment bundles. The hypothesis is that physician adherence to site-of-care decisions will be 20% higher in the intervention group.

C. Functional status (defined by SPI, activities of daily living and instrumental activities of daily living) and adverse events (that is the recurrence rate at 30 days, rate of complications (acute renal failure, ICU requirement, nosocomial infections or medication side effects), delirium, falls or pressure ulcers). Our hypothesis is that there is no difference in the functional status achieved 30 days after inclusion between the two groups.

D. Patient satisfaction with the provided service and quality of care at 30 days and 90 days.

E. Healthcare-related quality of life at the day of discharge as measured with the EQ-5D questionnaire. The hypothesis for D and E is that there is no difference in patient satisfaction and health-related quality of life between the intervention and the control groups.

F. We will describe the effective and chargeable costs for the entire treatment pathway in an exploratory economic analysis.

Endpoints will be assessed at hospital discharge, and 30 and 90 days after admission. An adverse event in a subject is defined as any untoward occurrence of any unfavorable and unintended clinically relevant medical sign, any symptom or any disease temporally associated with the study, which must not necessarily have a causal relationship with the study procedure. Any serious unexpected adverse event (for example, transfer to ICU, re-hospitalization, recurrence of an UTI, papillary necrosis, intrarenal or perirenal abscess, emphysematous pyelonephritis, allergic reaction to contrast medium during ultrasound or death) within 180 days after study inclusion is monitored by the data safety and monitoring board (DSMB). All adverse events will be followed until resolution, until the condition stabilizes, until the event is otherwise explained or the subject is lost to follow-up.

### Sample size

#### Intervention A

The first co-primary objective is to show that the duration of antibiotic therapy in the index hospitalization is 2 days shorter in the PCT group (8 days, standard deviation ±5) than in the control group (10 days, standard deviation ±5). These estimates are based on a historical control group in our hospital [31,36]. According to these estimations, 99 patients per arm would provide an 80% power at the 5% alpha level.

#### Intervention B

Similarly, for LOS estimation in patients with UTI we used data from a previous study performed on site at the Cantonal Hospital of Aarau during 2011, which approximated 6 days (standard deviation ±4) [36]. Our working hypothesis is that index hospitalization is 1.5 days shorter in the ProADM group (4.5 days, standard deviation ±4). According to these estimations, 112 patients per arm would provide an 80% power at the 5% alpha level (total sample size 224).

Based on previous research at the Cantonal Hospital of Aarau, there will be approximately 180 to 200 patients with UTI in a 12-month period, of which approximately 75 are expected to consent to this trial. Thus we estimate to complete the trial within 36 months.

The necessary sample sizes are shown in Table 2 assuming varying differences in LOS and antibiotic duration between the two intervention groups. Therefore, we will aim for a sample size of 250.

**Table 2:** Sample size considerations

**Time 1**	**Time 2**	**Power**	**Alpha-level**	**SD**	**Number per group**
10	8	0.8	0.05	4	63
10	8	0.8	0.05	5	99
10	8	0.8	0.05	6	142
10	9	0.8	0.05	4	252
10	9	0.8	0.05	5	393
10	9	0.8	0.05	6	566
6	4.5	0.8	0.05	4	112
6	4.5	0.8	0.05	5	175
6	4.5	0.8	0.05	6	252
6	5	0.8	0.05	4	252
6	5	0.8	0.05	5	393
6	5	0.8	0.05	6	566

### Statistical considerations

The primary analysis population will be the full analysis set, which includes all patients following an intention-to-treat principle. Every effort will be made to keep the number of losses to follow-up minimal. A sensitivity analysis will be carried out including only patients with evaluable primary endpoint data to assess potential bias due to patients lost to follow-up. Two co-primary endpoints will be used to compare, for Intervention A, the duration of antibiotic therapy and, for Intervention B, the length of physician-led hospitalization. A secondary analysis population, the per-protocol population, will be defined, which excludes major protocol violators. Specifically, patients will be excluded from the per-protocol population if they fulfill any of the following: violation of inclusion or exclusion criteria, not treated according to the trial requirements (that is, overriding of triage recommendations on admission or on day 3 without a pre-specified reason) or lost to follow-up. As a sensitivity analysis, the primary analysis will be repeated on the subset of patients with evaluable outcomes (that is, drop-outs are excluded from this additional analysis) and repeated on the per-protocol population.

To test the two continuous primary endpoints of this two-by-two factorial design study we will use the Mann–Whitney *U* test and calculate a median regression model [54]. We do not expect an interaction between the co-primary endpoints (for Intervention A the duration of antibiotic therapy and for intervention B the length of stay in our study). In our previous studies we showed that the shorter duration of antibiotic therapy in LRTIs did not influence the overall length of stay [41,55]. However, our algorithm for antibiotic guidance is different between inpatients and outpatients, which makes effect modification possible. Therefore we will formally test for an interaction using the median regression model. We are aware that the test for an interaction may result in a false negative due to the low power and the rather small sample size [56,57].

In a second step, the primary endpoint will be explored for association with potential prognostic factors in a median regression model considering potential confounding factors such as: age, sex, UTI subgroup, time to reach stability, co-morbidities, patient preferences and living conditions prior to hospitalization.

#### Analysis of secondary endpoints

For both interventions, the secondary endpoints will be compared by the *t*-test if normally distributed, by the Mann–Whitney *U* test if similarly shaped but are not normally distributed continuous data, by chi-square tests for categorical data or by the log-rank test for time-to-event data, as appropriate. Since this is a feasibility study we will not have enough power to test for non-inferiority. Estimates of effect size and corresponding confidence intervals will be provided. Exploratory analyses of endpoints adjusted for possible confounders will be performed using linear or logistic regression or Cox proportional hazards models, as appropriate. Prognostic accuracy will be assessed by receiver operating characteristic curve analysis and by calculating sensitivity, specificity, positive and negative predictive values and likelihood ratios for medical and biopsychosocial risk assessments.

## Discussion

The results of earlier studies by our group provided evidence that antibiotic therapy can be guided in patients with LRTI using PCT cutoff ranges. We recently showed that biomarkers, especially ProADM, are able to predict the outcome for patients with LRTIs [58]. On account of this, we added the ProADM to the widely used CURB65 score, resulting in the CURB65-A score capable of guiding triage decisions [31,35].

In this study we extend our concepts to patients with UTI, which contributed approximately 7% of patients in the previous French multi-center ProRATA study [13]. UTIs, as one of the most common indications for antibiotic therapy [59], provide an ideal opportunity for extrapolating our knowledge from LRTI studies.

In the current guidelines [4,10,19] a crucial point in the determination of the length of antibiotic therapy and whether a patient has to be hospitalized or not, has been the distinction between lower and upper UTI, and this classification is still based on subjective criteria. The first aim of this study is to create evidence-based guidelines, which will allow clinicians to administer tailored antibiotic therapy in uncomplicated as well as in complicated UTIs by using PCT levels and pyuria dynamics. This will help to determine the optimal length of antibiotic therapies and avoid antibiotic overuse.

As shown in a study among a population of frail elderly nursing home residents, 43% of all patients receiving antibiotic treatment for an assumed UTI are over-diagnosed and actually do not require antibiotics [60]. Another large potential for reducing antibiotic use lies in febrile UTI patients, who currently receive at least 7 days of antibiotics according to guidelines.

The second aim is to support clinicians in site-of-care decision-making, for which we will use the ProADM cutoff levels we extracted from our previous trials [29,31,35,36]. This study can provide proof of our concept, in that the algorithms we obtained in our LRTI studies can be adapted to other common infections with only minor disease-specific adaptations. Given the fact that UTIs have a high prevalence, any improvement to the current risk assessment is of both great socio-economic and scientific interest. Hospital stays are very costly and avoiding or shortening the LOS could result in large savings [61]. Clinical severity scores classify patients according to the presence of co-morbidities and subjective criteria [45]. Even though recommended in guidelines, these clinical scores have the disadvantage of being too general and do not give sufficient weight to the host response [50,62]. Adding objective criteria such as easily measurable biomarkers to these clinical scores individualizes medical decisions and might detect subgroups that have a higher risk of suffering complications and therefore require increased attention and particular care. On the other hand, a trustworthy method of identifying patients with a low risk of adverse events, allows outpatient treatment with lower risk of healthcare-associated infections [63], as well as treatment in nurse-led care. It gives physicians, nurses, patients and relatives the required confidence in their decisions. Risk-based triage could reduce bed shortages during peak times and lead to lower costs.

### Potential limitations

This protocol describes two randomized open intervention trials in one factorial study design with an expected high external validity. However, contamination within the proposed open trial design is obvious. The Hawthorne effect of an earlier discharge in the intervention group compared to the control group is possible as well as a spillover effect since PCT and ProADM values are not blinded because they are part of the routine chemistry. However, the bias due to the Hawthorne effect for the primary endpoints (for Interventions A and B) is expected to be conservative and favor the null hypothesis. The research team emphasizes treatment and triage algorithms according to the randomized group. Previous studies showed poor guideline compliance despite high-intensity implementation. Therefore the expected spillover effect will also have a conservative influence on the primary endpoints. We minimize ascertainment bias by having objective, measurable primary endpoints. More subjective secondary endpoints will be assessed by blinded researchers.

## Abbreviations

Abx: Antibiotics; CAP: Community acquired pneumonia; CEUS: Contrast-enhanced ultrasound; cfu: Colony-forming unit; CRP: C-reactive protein; DSMB: Data safety and monitoring board; ED: Emergency department; EDTA: Ethylenediaminetetraacetic acid; GFR: Estimated glomerular filtration rate with MDRD formula; ICU: Intensive care unit; LOS: Length of stay; LRTI: Lower respiratory tract infection; NSAIDs: Nonsteroidal anti-inflammatory drugs; PACD: Post-acute care discharge score; PCT: Procalcitonin; ProADM: Proadrenomedullin; PSA: Prostate-speicifc antigen; SPI: *Selbstpflegeindex* self-care deficit score; TMP-SMX: Trimethoprim-sulfamethoxazole; UST: Urinalysis; UTI: Urinary tract infection

## Competing interests

This is an investigator-initiated study. To exclude any conflict of interest, no commercial sponsor has any involvement in design and conduct of the trial, that is, collection, management, analysis and interpretation of the data and preparation, decision to submit, review or approval of the manuscript. For other studies unrelated to this trial Werner Albrich, Philipp Schuetz and Beat Müller received support from BRAHMS Thermo Fisher and from bioMérieux to attend meetings and fulfill speaking engagements and served as consultants for BRAHMS Thermo Fisher. Beat Müller received research support from BRAHMS Thermo Fisher. No other authors have disclosed any conflicts of interest.
